# Facile Synthesis of Monodisperse Gold Nanocrystals Using *Virola oleifera*

**DOI:** 10.1186/s11671-016-1683-3

**Published:** 2016-10-18

**Authors:** Bárbara A. Milaneze, Jairo P. Oliveira, Ingrid Augusto, Wanderson J. Keijok, Andressa S. Côrrea, Débora M. Ferreira, Otalíbio C. Nunes, Rita de Cássia R. Gonçalves, Rodrigo R. Kitagawa, Vinícius G. Celante, André Romero da Silva, Ana Claudia H. Pereira, Denise C. Endringer, Ricardo P. Schuenck, Marco C. C. Guimarães

**Affiliations:** 1 Federal University of Espirito Santo, Av Marechal Campos1468, Vitória, ES 29.040-090 Brazil; 2 Federal Institute of Espírito Santo, Av. Morobá, 248 - Morobá, Aracruz, ES 29192-733 Brazil; 3 Vila Velha University, Rua Comissário José Dantas de Melo, 21, Boa Vista, Vila Velha, ES 29102-770 Brazil

**Keywords:** Gold nanoparticles, Green synthesis, *Virola oleifera*, Flavonoids

## Abstract

The development of new routes and strategies for nanotechnology applications that only employ green synthesis has inspired investigators to devise natural systems. Among these systems, the synthesis of gold nanoparticles using plant extracts has been actively developed as an alternative, efficient, cost-effective, and environmentally safe method for producing nanoparticles, and this approach is also suitable for large-scale synthesis. This study reports reproducible and completely natural gold nanocrystals that were synthesized using *Virola oleifera* extract. *V. oleifera* resin is rich in epicatechin, ferulic acid, gallic acid, and flavonoids (i.e., quercetin and eriodictyol). These gold nanoparticles play three roles. First, these nanoparticles exhibit remarkable stability based on their zeta potential. Second, these nanoparticles are functionalized with flavonoids, and third, an efficient, economical, and environmentally friendly mechanism can be employed to produce green nanoparticles with organic compounds on the surface. Our model is capable of reducing the resin of *V. oleifera*, which creates stability and opens a new avenue for biological applications. This method does not require painstaking conditions or hazardous agents and is a rapid, efficient, and green approach for the fabrication of monodisperse gold nanoparticles.

**Graphical Abstract:**
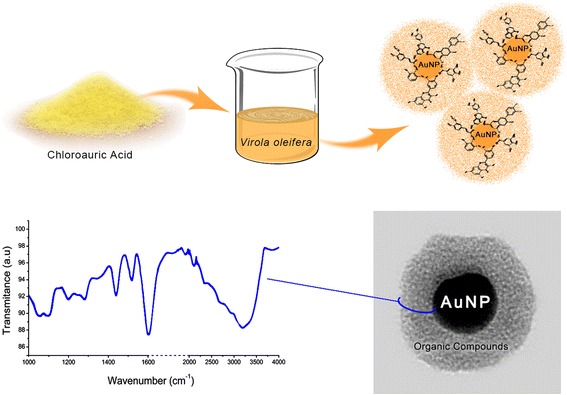
The *Virola oleifera* reduction method for the synthesis of gold nanoparticles (AuNP’s)

The *Virola oleifera* reduction method for the synthesis of gold nanoparticles (AuNP’s)

## Background

The emergence of nanotechnology has significantly expanded the applications of nanoparticles by allowing intimate interfaces among materials science, chemistry, physics, and biology to better manipulate and utilize their properties [1, 2]. Several current studies have been devoted to the synthesis, stabilization, and functionalization of gold nanoparticles (AuNPs) [3, 4].

The interest in AuNPs is largely due to the relative ease of their synthesis, which offers good control of their sizes and shapes as well as their optical characteristics. Moreover, the versatile surface chemistry of gold nanoparticles allows them to be coated with a wide range of molecules [5, 6]. Therefore, various applications [7–12] have been integrated into different areas to enable the development of new manufacturing processes [13, 14].

Recently, structural studies of nanoparticles have indicated that chemical synthesis can be replaced by an environmentally friendly process using plant extracts [15–20], in which nanoparticles that are traditionally synthesized in a chemical process are attached to and subsequently released from rigid delivery substrates for biomedical applications [21, 22]. Therefore, the main roles of the plant extract are to act as reducing and capping agents during the synthesis process [23]. Moreover, plant extracts appear to be the best route for the large-scale synthesis of nanoparticles. The green growth of nanoparticles depends on many polyphenol quantities [24]. In this study, gold nanoparticles were synthesized using a *V. oleifera* extract*.*

*V. oleifera* offers numerous distinct advantages, such as biocompatibility, non-toxicity, aqueous solubility, and their beneficial composition that is rich in phenols (i.e., ferulic and gallic acid, epicatechin, tannins, and quercetin) [25].

Here, we have investigated the design of a new route for the production of gold nanoparticles using a vegetable extract, and the AuNPs produced in this study were distinct from those previously reported [26, 27]. Our method offers several advantages in terms of easy scale-up, environmental friendliness, and low cost. We believe that the broad application of this strategy will contribute to the development of the next generation of green nanotechnology.

## Methods

### Plant Material

*Virola oleifera* (Schott) A. C. Smith is grown in the Atlantic forest in the southeastern region of Brazil. The plant material was verified by D.Sc. Luciana Dias Thomaz from the Department of Botany, Federal University of Espírito Santo, where the voucher specimen was deposited (VIES 19648). The fluid exudate was obtained by creating a 0.5-m incision in the tree trunk and collecting the resin in an aseptic plastic container, and this resin was transferred to an amber glass vial that was stored at 4 °C prior to analysis. Then, the fluid exudate was subjected to drying at 40 °C followed by grinding to yield 24 g of dried resin.

### Experimental Design and Statistical Analysis

Two variables that influence the nanoparticle size and concentration were evaluated in this study including the reducing agent concentration and synthesis time. To reduce the number of process variables, the concentration of the gold precursor agent was fixed. A 3^2^ factorial design with 3 levels and 2 variables (Table 1) was assembled to verify its influence on the conversion of the reaction and to determine the optimal conditions for nanoparticle synthesis.

**Table 1:** Experimental design

Variables	Levels		
	Low	Medium	High
Volume of *Virola oleifera*	1 mL	2 mL	3 mL
Time of the synthesis	10 min	20 min	30 min

In this study, the following concentrations were employed: 1 g/L *Virola oleifera* and 2.5 × 10^−4^ mol/L tetrachloroauric acid trihydrate (HAuCl_4_·3H_2_O). Ten milliliters of a gold solution was used in all of the experiments

The effects of each selected variable on the nanoparticle size were analyzed using STATISTICA version 10.0. The inputs included the absorbance values of the as-prepared samples at 530 nm, and the corresponding diameters were calculated using a trial version of the ImageJ software. An analysis of variance (ANOVA) of the data was also performed, and the values were considered significant at *p* values <0.05. The optimal values of the independent variables were determined using a three-dimensional analysis of the response surface of the independent and dependent variables.

### Gold Nanoparticles Synthesis

To produce AuNPs, we performed a 3^2^ factorial design with 3 levels and 2 variables (Table 1) to investigate the cause and effect relationship between the reducing agent and the time. For the gold nanoparticle synthesis, a 2.5 × 10^−4^ M gold chloride solution (Sigma-Aldrich, St Louis, MO, USA) was employed, and three percentages of the reducing agent (*V. oleifera*) were added according to the experimental plan. The plant extract was added to the gold precursor solution. The mixture was maintained at room temperature (25 °C) under stirring (200 rpm) for three time periods (Table 1) and protected from light. The AuNP formation was observed by the appropriate color change.

### Electrochemical Measurements

The electrochemical measurements were carried out using a Metrohm Autolab PGSTAT 128n instrument. Three electrodes were employed. The working electrode was Pt (0.5 cm^2^), a leak-free saturated Ag/AgCl/KCl electrode was employed as the reference electrode, and the counter electrode consisted of a Pt plate (3.0 cm^2^).

### Characterization of Gold Nanoparticles (UV-Vis, TEM, DLS, SEM, HR-SEM, FTIR, Raman, and X-ray)

A UV-visible spectrophotometer (UV-1800 Shimadzu, Japan) was used to determine the surface plasmon resonance absorption (SPR) by scanning from 400 to 700 nm. For transmission electron microscopy (TEM) visualization, AuNPs were mounted on formvar-coated 200 mesh grids (Ted Pella Inc., USA), negatively stained using an 5 % aqueous uranyl acetate solution (Sigma-Aldrich) and visualized using a transmission electron microscope JEM-1400 (JEOL, São Paulo-SP, Brazil) at 120 kV with lab6 filament. The particle size distribution and zeta potential were determined using dynamic light scattering (DLS) technology (NPA152 Zetatrac, Microtrac Instruments, York, USA) combined with the interaction of random Brownian motion and the driven electric field motion of the particle suspensions. The total mass of the recovered nanoparticles that were obtained after the washing step was suspended in 50 mL of deionized water and sonicated at 50 Hz for a period of 1 min. The zeta potential values represent the average standard deviations for three independent preparations of nanoparticles. The total AuNP concentration was determined using ICP-MS (Perkin Elmer, Optima 7000, USA). The surface, shape, and dispersity characteristics were determined by SEM (JEM 6610-LV, JEOL, São Paulo-SP, Brazil) and HRSEM (Auriga, Zeiss, German). The monodispersity of the AuNPs was determined based on the aspect ratio (length to diameter, L:D) using the ImageJ software (*N* = 200 nanoparticles). After the AuNP synthesis, the samples were centrifuged at 15,000 rpm for 30 min and freeze-dried using a speed vac. A Fourier transform infrared spectroscopy (FTIR) analysis was performed in ATR mode (FT-MIR FTLA 2000 Bomem) to investigate the interactions between the organic compounds of the *V. oleifera* extract and the nanoparticles, and the functionalized AuNPs were characterized by Raman spectrometry (ALPHA 300R Confocal Raman Spectrometer) in the 500–3500 cm^−1^ region at a laser power of 532 nm. Finally, the crystal structure was determined using X-ray diffraction (XRD). The AuNP samples were prepared by drop-coating the pelletized AuNPs on a glass slide and scanning in a 2θ region from 30° to 90° at 0.01° per minute with a time constant of 2 s using a D8 Advance (Bruker-axs) X-ray diffractometer.

## Results and Discussion

### Fabrication of Colloidal Gold Nanoparticles and Effects of Two Parameters on AuNP Formation

The influence of the volume of the *V. oleifera* extract and the reaction time on the formation of AuNPs was evaluated to determine the optimal conditions for this synthesis. We applied a 3^2^ factorial design that was performed by varying the values of the studied parameters, as shown in Table 1. The details of the experimental procedures and characterizations are provided in the “Methods” section.

**Fig. 1:**
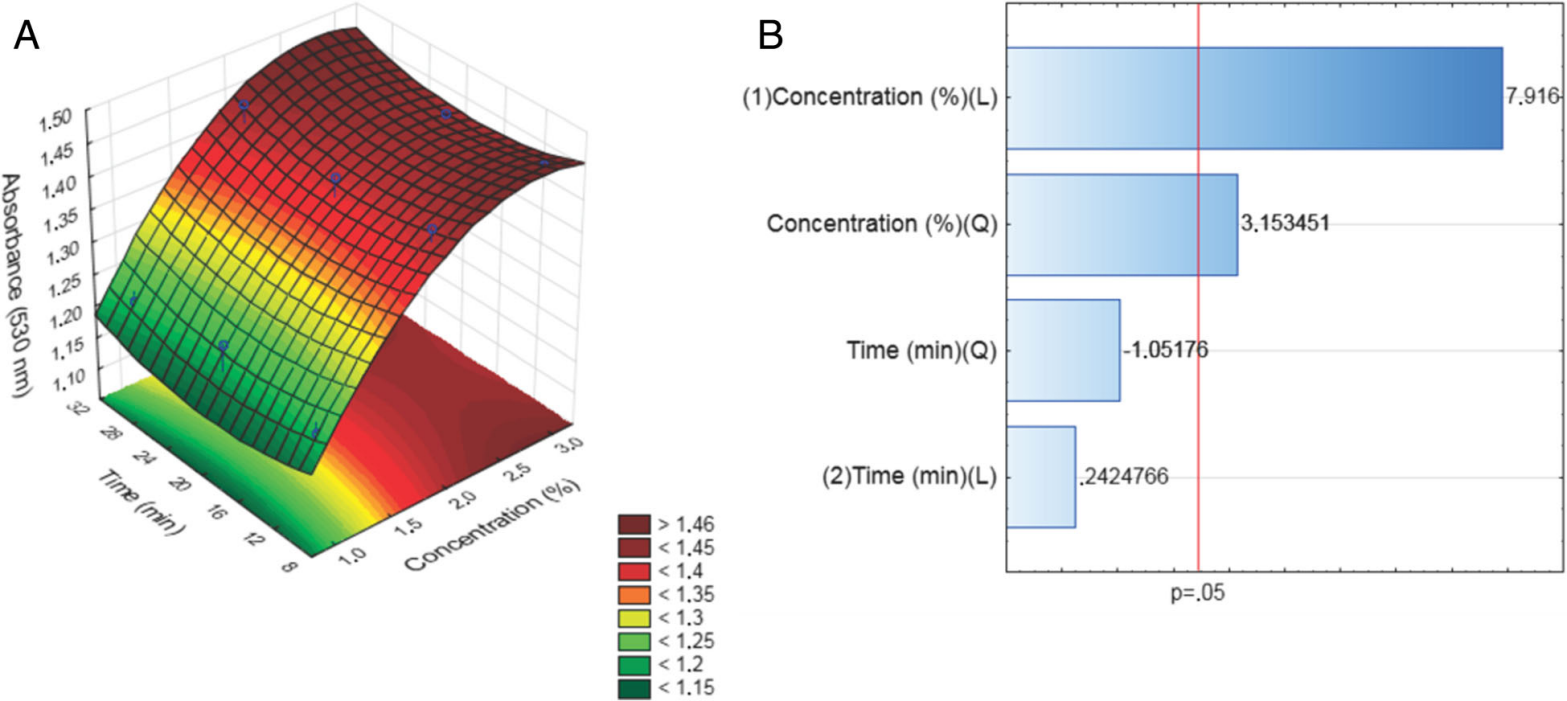
**a** Response surface curve and **b** standardized Pareto chart for the effect of the volume of *Virola oleifera* and the reaction time on the AuNP formation. The *bars* that exceed the vertical line on the graph indicate that the corresponding factor terms are significant (*p* < 0.05)

A three-dimensional plot and contour map for −log(*N*/*N*_0_) of the absorbance for the AuNP synthesis as a function of time and the volume of the *V. oleifera* extract was obtained using response surface modeling (RSM). The results indicated that an increase in the volume of the *V. oleifera* extract caused an increase in the absorbance value, suggesting an increase in the number of AuNPs (Fig. 1a). However, the same influence on the AuNP formation was not observed as the reaction time increased.

The relative contributions from the concentration and time to the AuNP growth are presented in a Pareto chart (Fig. 1b). When we independently analyzed the variables, only the concentration resulted in a significant effect (value 7.916) (Fig. 1b). No significant combined effect was observed for the preparation of the nanoparticles. The relative importance of the independent variables was specified by the standardized main effect of the Pareto chart (Fig. 1b). The significance was based on the *F* values and *P* values. A larger *F* value and a smaller *P* value indicate a more significant variable and corresponding coefficient.

### Electrochemical Performance of AuNPs Versus *V. oleifera* Extract

**Fig. 2:**
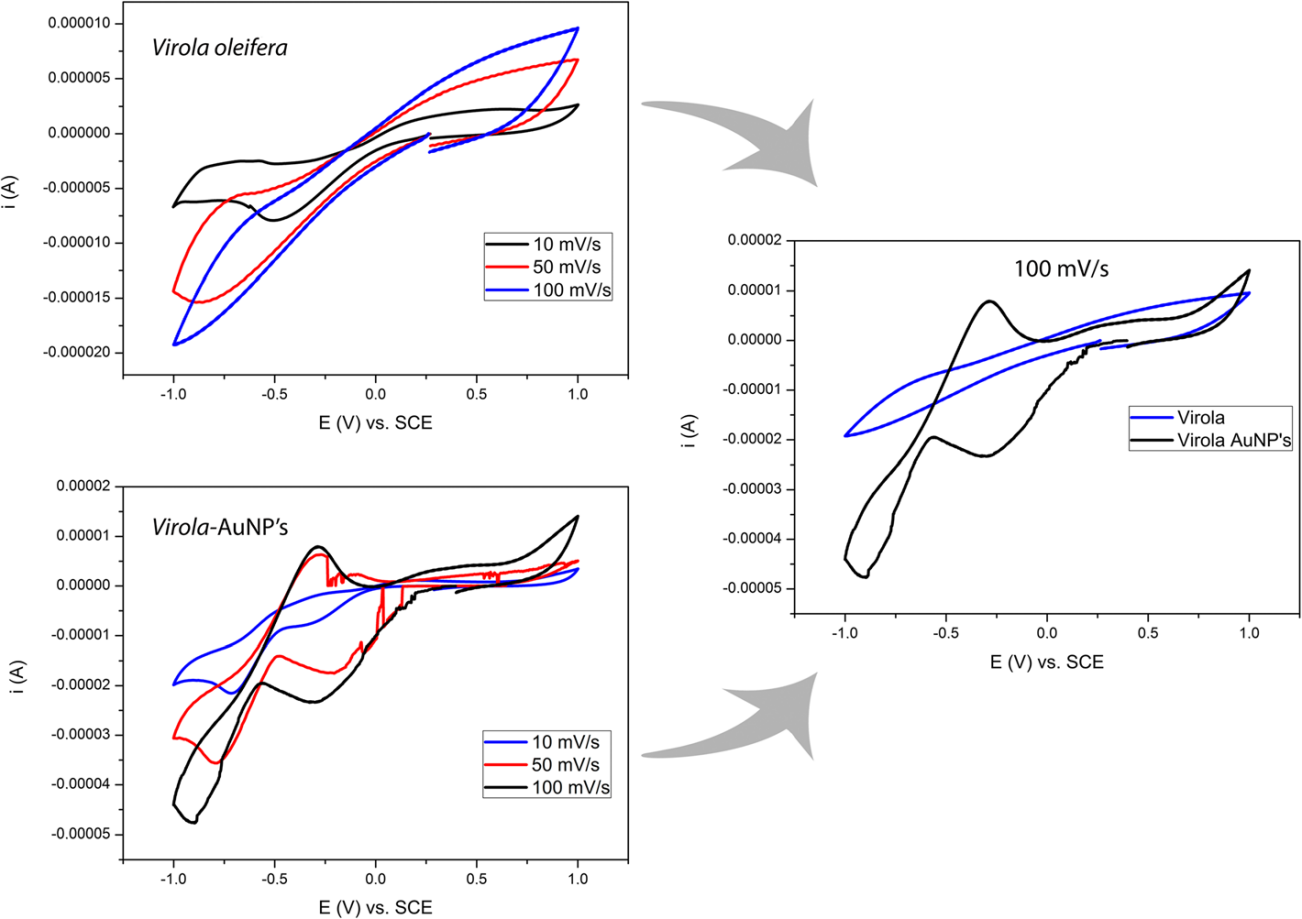
Cyclic voltammograms of *Virola oleifera* (i.e., scan recorded at 10, 50, and 100 mV/s). Cyclic voltammetry of colloidal gold nanoparticles synthesized with *V. oleifera* at different scan rate (10, 50, and 100 mV/s). Comparison between *V. oleifera* and colloidal gold nanoparticles at 100 mV/s

To evaluate the role of *V. oleifera* in the AuNP synthesis, the electrooxidation of the *V. oleifera* extract was performed. We compared the cyclic voltammetry of the *V. oleifera* extract and the AuNPs at three different scanning speeds (i.e., 10, 50, and 100 mV/s) (Fig. 2). The current and potential reduction in the *V. Oleifera* system could not be accurately determined (Fig. 2a) because it had not been oxidized. The AuNPs that were synthesized using *V. oleifera* (Fig. 2b) exhibited more defined cathodic regions due to the system being previously oxidized. In addition, an oxidation peak was observed in the region close to −0.25 V.

This process may be related to the initial oxidation. To compare *V. oleifera* and AuNPs, we focus on the results using a scan rate of 100 mV/s (Fig. 2c). Notably, the gold system undergoes a reduction process that is much more defined. However, the system containing only *V. oleifera* does not exhibit the same characteristic due to a lack of previous oxidation.

### Characterization of Gold Nanoparticles

**Fig. 3:**
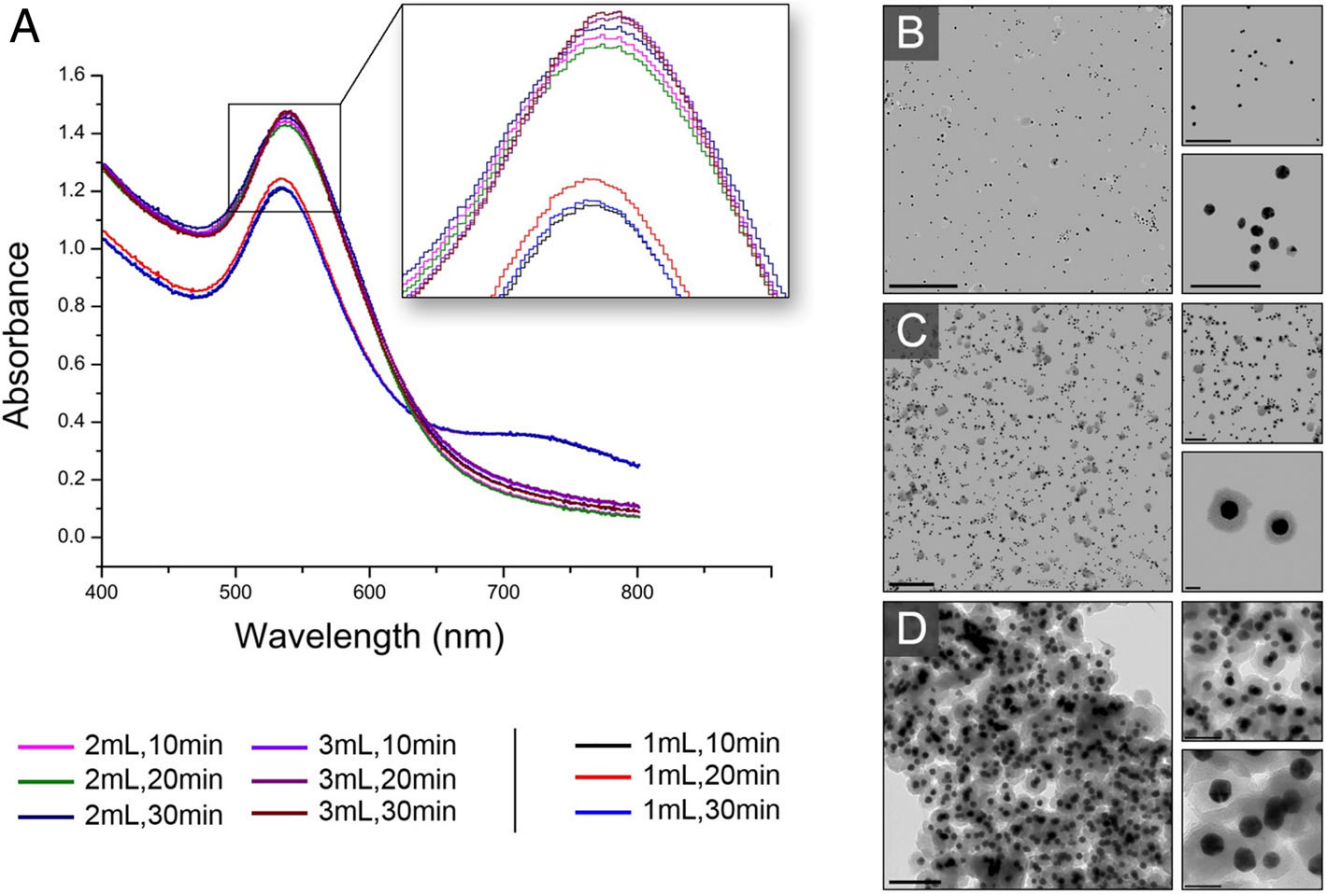
**a** UV-visible spectra of gold nanoparticles (AuNPs) under different conditions. Note the plasmon peak in the inset. **b**–**d** TEM images of AuNPs produced using 1, 2, and 3 mL of *Virola oleifera*, respectively, and a reaction time of 20 min. **b** TEM images of AuNPs produced with 1 mL and 20 min. (The *scale bars* are 0.5 μm for the enlarged image, 0.2 μm for the top image, and 100 nm for the bottom image.) **c** TEM images of AuNPs produced with 2 mL and 20 min. (The *scale bars* are 0.5 μm for the enlarged image, 0.2 μm for the top image, and 20 nm for the bottom image.) **d** TEM images of AuNPs produced with 3 mL and 20 min. (The *scale bars* are 200 nm for the enlarged image, 100 nm for the top image, and 50 nm for the bottom image)

**Fig. 4:**
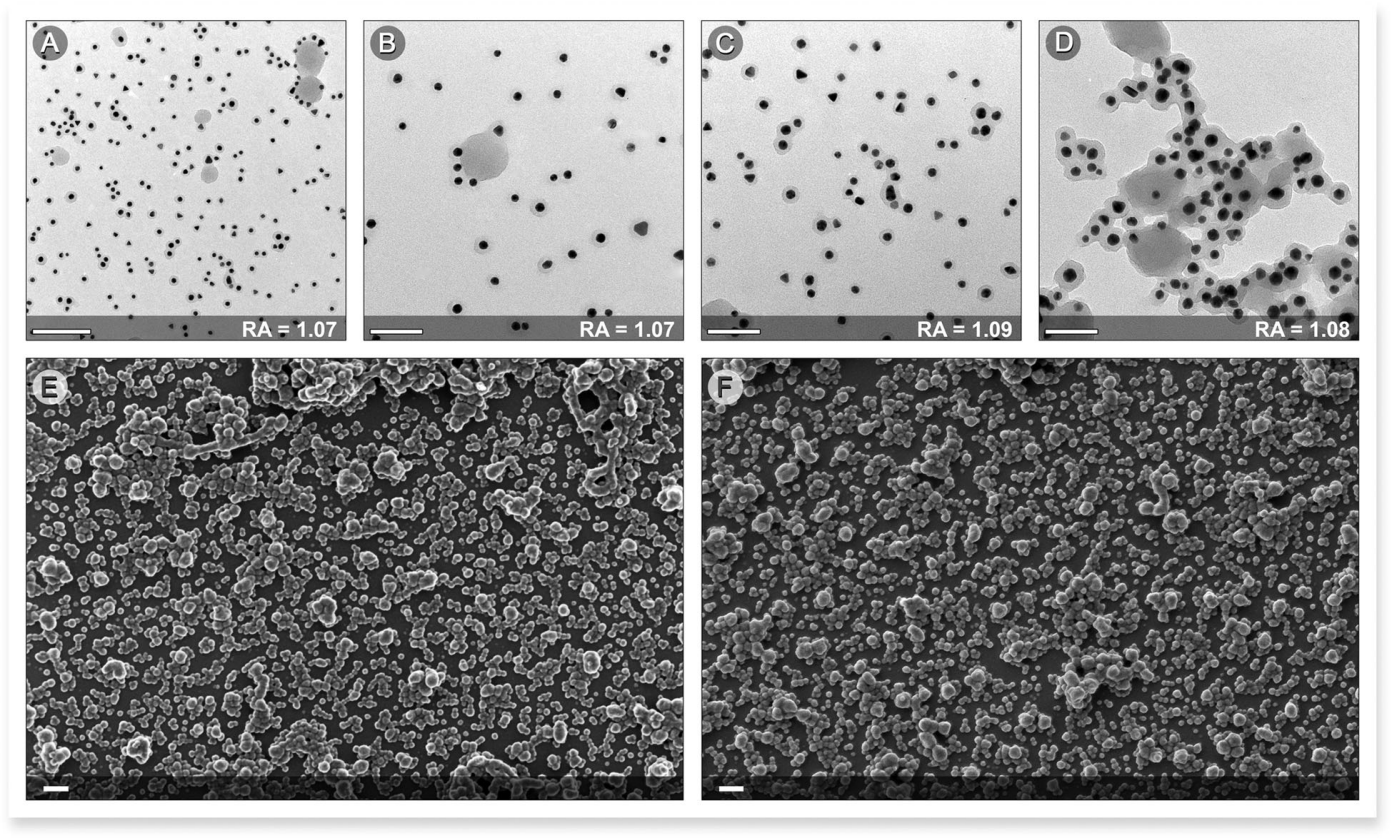
TEM images of AuNPs used to determine the monodispersity (**a**–**d**) with the aspect ratio (AR) value inset. The *scale bars* represent **a** 200 nm and **b**–**d** 100 nm. HR-SEM images of the AuNPs (**e**, **f**), and the *scale bars* represent 200 nm

The transmission electron microscopy results indicate that the Au-synthesized nanoparticles were typically spherical in shape and monodisperse (Figs. 3b–d and 4). To examine the growth, size, and shape of the AuNPs in aqueous suspensions by surface plasmon resonance (SPR), a UV-visible spectrophotometer was employed. The UV-visible spectra of the reaction solutions exhibited a characteristic surface plasmon resonance band corresponding to the AuNPs at approximately 539 nm under all of the conditions (Fig. 3a). The sharpness of the UV peak also indicates that the sizes of the AuNPs in these samples were nearly monodisperse. The size of the nanoparticles can be monitored indirectly by the integrated area of the suspension absorbance spectra (AISAS) in the region where no AuNP absorbance was observed. Because the light scattering for a nanoparticle is proportional to the square of the particle volume, a suspension with smaller particles scatters less light, and its absorbance spectrum has a smaller integrated area [28]. The AISAS increased from 49 to 55 and 58, and the volume of the *V. oleifera* extract increased from 1 to 2 and 3 mL, respectively, for a reaction time of 20 min. The same results were observed for the other reaction times. This result indicated that an increase in the extract volume resulted in an increase in the AuNP size, which was most likely due to the adsorption of a greater amount of *V. oleifera* on the AuNP surface, which increased the nanoparticle size. This result was confirmed by monitoring the average size of the nanoparticles using dynamic light scattering measurements because the average size increased from 1.1 to 43 and 73 nm when the volume changed from 1 mL to 2 mL and 3 mL, respectively.

Gold nanoparticle properties are dependent on their dimensions. Therefore, a monodispersity analysis is important for evaluating the nanoparticles’ synthesis and application. The aspect ratio (D/L) was calculated. As observed in the TEM images (Fig. 4a–d) and confirmed by HR-SEM (Fig. 4e, f), the resulting particles are relatively monodisperse under these nucleation and growth conditions.

The amount of Au present in the AuNP nanoparticle was quantified using inductively coupled plasma mass spectrometry (ICP-MS). The ICP-MS analysis indicated that the total amount of AuNPs changed from 60.0 to 49.9 and 42.8 mg/L when the synthesis was performed with 1, 2, and 3 mL of the *V. oleifera* extract, respectively. The adsorption of a larger amount of *V. oleifera* extract on the nanoparticle surface may limit the increase in the amount of Au in the nanoparticle.

**Fig. 5:**
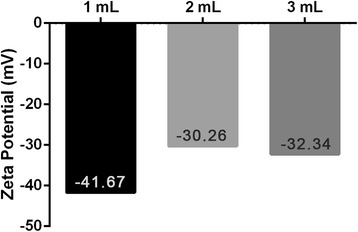
Surface charge measurements based on the zeta potential of the AuNPs under different conditions. Measurements of the zeta potential of the AuNPs at various *Virola oleifera*/HAuCl_4_·3H_2_O ratios

The colloidal stability of the AuNPs was initially confirmed by measuring the zeta potential to estimate the charge on the nanoparticle surface. The zeta potential analysis revealed that the charge on the AuNP surface decreased from −41.67 to −30.26 and −32.34 mV as the volume of the *V. oleifera* extract increased from 1 to 2 and 3 mL, respectively (Fig. 5). This result confirmed that the extract was adsorbed on the nanoparticle surface, which decreased the zeta potential. The AuNPs synthesized with 1 and 2 mL of *V. oleifera* generated stable nanoparticles in distilled water. In general, nanoparticles with a zeta potential greater than +25 mV or less than −5 mV possess sufficient electrostatic repulsion to remain stable in solution [29]. A larger negative value may be due to the capping layer of the *V. oleifera* composition. The repulsive forces between negatively charged particles prevent agglomeration.

**Fig. 6:**
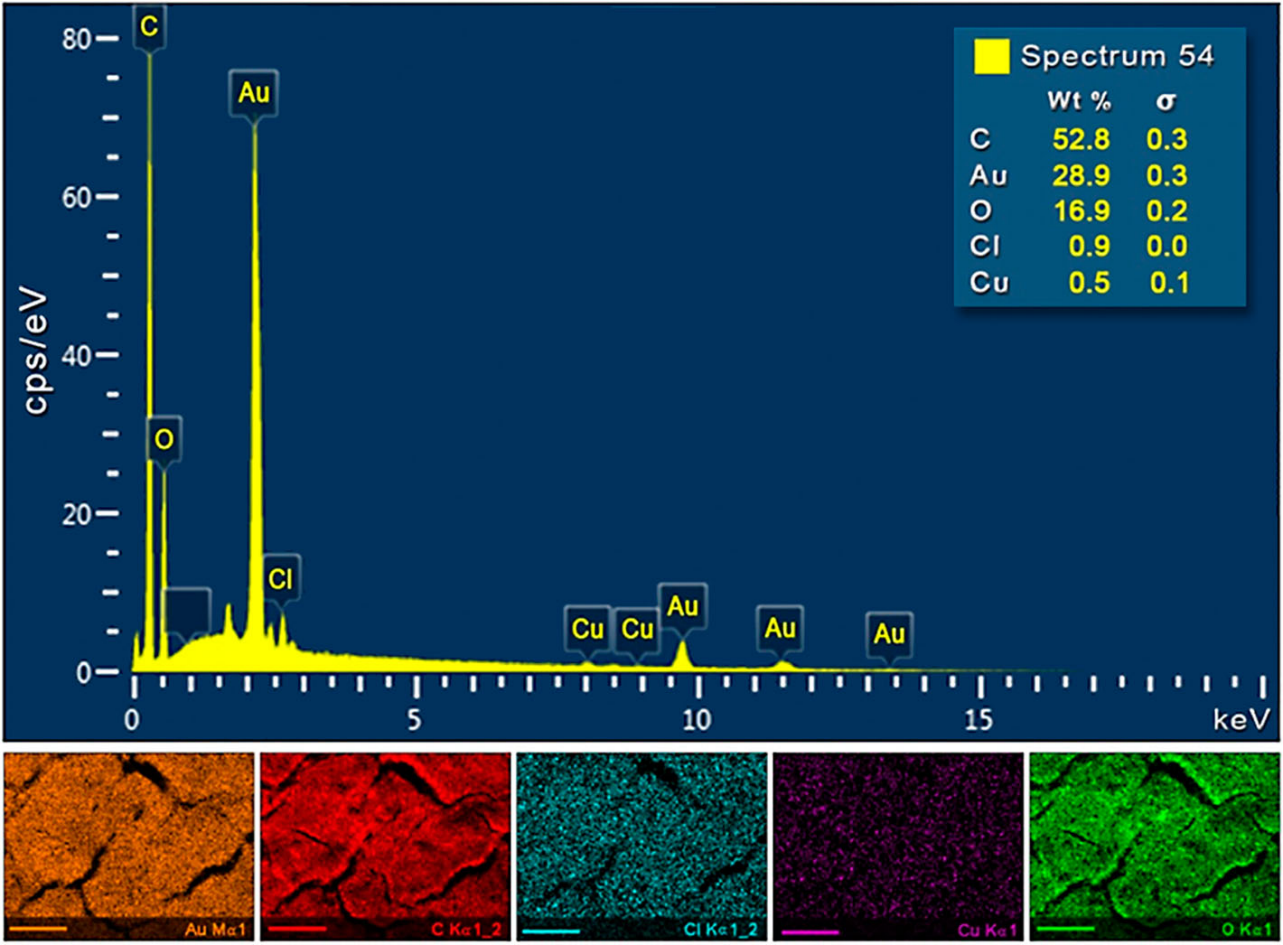
SEM images and corresponding energy-dispersive X-ray spectroscopy (EDS) elemental maps of the AuNPs spectra collected at the numbered sites indicated on the micrographs. Different colors and intensities represent the concentration of these elements. The *scale bars* represent 2 μm

An aliquot of the sample synthesized with the intermediate conditions of the experimental design (i.e., synthesis time of 20 min and *V. oleifera* volume of 2 mL) were characterized to better understand the composition of the as-synthesized nanoparticles. Energy-dispersive X-ray spectroscopy (EDS) (Fig. 6) was performed to confirm the composition of the AuNPs and the presence of other elements. The more intense Au peaks were acquired at approximately 2.3 Kev for Virola-AuNPs. The elemental mapping was performed for a selected region (5 × 5 μm^2^) and indicated a high deposition of carbon (red), gold (orange), and oxygen (green) with the presence of other elements (i.e., copper (purple) and chlorine (cyan)).

**Fig. 7:**
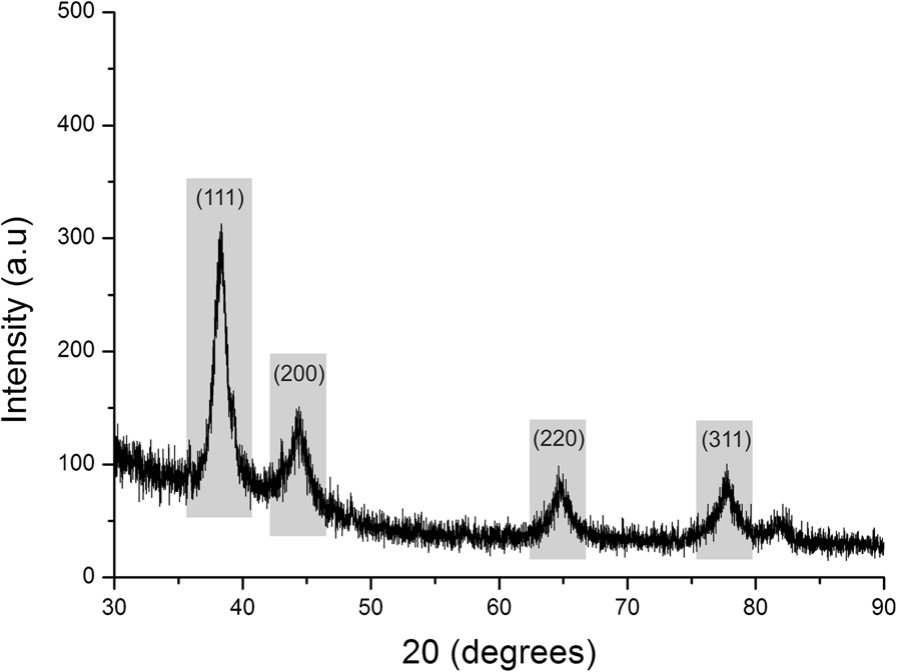
Powder X-ray diffraction pattern for a gold nanoparticle prepared with 2 mL of *Virola oleifera* extract over 20 min. Broad peaks were observed and are characteristic of nanocrystals. The Au 111 peak at 38.1° was very intense due to the orientation of the Au

Finally, the crystalline nature of the biosynthesized gold nanoparticles was further confirmed from XRD analysis. The XRD patterns of the synthesized gold nanoparticles are shown in Fig. 7. Four intense diffraction peaks were observed at 2θ values of 38.31°, 44.45°, 64.64°, and 77.73°, corresponding to the (111), (200), (220), and (311) reflections of metallic crystalline gold, respectively. A strong diffraction peak corresponding to the (111) facet was observed, and the peaks corresponding to the three other facets were less intense. This observation suggested that the (111) plane was the predominant orientation of the as-prepared AuNPs using the *V. oleifera* extract.

### Presence of Phenolic Compounds on the Surface of the AuNPs Synthesized with *V. oleifera* Ace

**Fig. 8:**
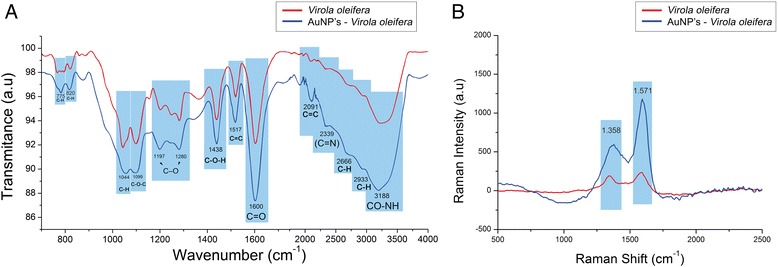
Relative infrared spectra of the *Virola oleifera* extract and AuNPs synthesized with *V. oleifera* under different conditions. *V. oleifera* is in *red*, and the AuNPs are in *blue* (**a**). Raman spectra of the *V. oleifera* extract, and AuNPs recorded at 532 nm (**b**)

Phenolic compounds possess biologically beneficial activities and are frequently utilized as antioxidants. To confirm the presence of macromolecules that play a key role in biological activity on the surface of the as-synthesized AuNPs, FTIR and RAMAN measurements were performed on both the nanoparticles and the pure extract. Infrared spectrometric analysis was applied to determine whether the nanoparticle modifies the chemical profile of the *V. oleifera* resin (Fig. 8a). The pure *V. oleifera* extract (in red) exhibited two strong characteristic bands. The band at approximately 1600 cm^−1^ that corresponds to a C=O stretching vibration [30] is associated with carbonyl bonding from a component of the *V. oleifera* extract (e.g., ferulic and gallic acid, epicatechin, tannins, and quercetin). Its components are typically observed between 1590 and 1820 cm^−1^ or 3000 and 3400 cm^−1^, which are characteristic of OH vibrational stretching [31, 32]. Moreover, less intense peaks were observed at 775 and 820 cm^−1^ (benzene ring vibrations) [30], 1044 cm^−1^ (C–H) [33], 1099 cm^−1^ (C–O–C) [34], 1197–1280 cm^−1^ (C–O) [35], 1438 cm^−1^ (C–O–H) [36], and 1517 cm^−1^ (C=C) [37]. The same spectral profile was observed for the AuNPs, suggesting that the *V. oleifera* resin was linked with the nanoparticles. Moreover, the shifts in peak positions between the extract and the AuNPs spectra may indicate bonding to the metal surface.

To investigate the Raman scattering performance, the signal of the AuNPs coated with organic compounds from *V. oleifera* was measured (with 633-nm laser excitation at 17 mW for 1 s) (Fig. 8b). To analyze the AuNP-associated biomolecules, two characteristics are extremely important: similarity and intensity. The *V. oleifera* and AuNPs-*V. oleifera* spectra were similar, and the spectrum of a molecule in contact with the nanocrystal was enhanced, which indicated that the compounds present in the plant extract functionalized the nanoparticles via irreversible electrostatic interactions. The Raman spectra for the HAuCl_4_ incubation conditions contained two very strong peaks at 1358 and 1571 cm^−1^. The position peaks corresponding to the symmetric and asymmetric stretching carboxylic groups (COO^−1^) are related to the peaks observed at 1358 and 1571 cm^−1^, respectively [38]. The observed vibrations have also been reported for free and Na feluric acid salt [39].

## Conclusions

The green synthesis of biofunctionalized AuNPs from *V. oleifera* was simple, environmentally friendly, and economical. Due to the reducing and capping nature of the bioactive compounds present in the aqueous extract of *V. oleifera*, a cap was formed around the gold ions of the stable biofunctionalized NPs. The presence of the functional group of the bioactive compounds was confirmed by FTIR and Raman spectroscopy. The particle size, quasi-spherical shape, and monodispersity of the AuNPs were determined by TEM, SEM, HRSEM, and XRD analyses. *V. oleifera* may serve as a source of biofunctionalized NPs for a wide range of in vitro assays. The results of this study are as follows: (i) the synthesis using *V. oleifera* allows for the preparation of monodisperse AuNPs that are homogenous in size and shape and stable in solution, (ii) the presence of organic compounds that can potentially offer important biological activity for these nanoparticles, and (iii) a new route for the production of bioactive gold nanoparticles.

Our studies provide an important basis for the application of the green synthesis of gold nanoparticles using *V. Oleifera*.
